# Mortality and Cardiovascular Complications in Older Complex Chronic Patients with Type 2 Diabetes

**DOI:** 10.1155/2017/6078498

**Published:** 2017-08-10

**Authors:** J. L. Clua-Espuny, M. A. González-Henares, M. L. L. Queralt-Tomas, W. Campo-Tamayo, E. Muria-Subirats, A. Panisello-Tafalla, J. Lucas-Noll

**Affiliations:** ^1^Department of Research, ICS Terres de l'Ebre, University Institute in Primary Care Research Jordi Gol (IDIAP Jordi Gol), Barcelona, Spain; ^2^EAP-Camarles-Aldea-Ampolla, Catalonian Health Institute, SAP Terres de l'Ebre, Health Department, Generalitat de Catalunya, CAP Ampolla, 43895 Tortosa, Spain; ^3^EAP Tortosa 1-Est, Catalonian Health Institute, SAP Terres de l'Ebre, Health Department, Generalitat de Catalunya, CAP Temple, 43500 Tortosa, Spain; ^4^EAP Tortosa-2-Oest, Catalonian Health Institute, SAP Terres de l'Ebre, Health Department, Generalitat de Catalunya, CAP Xerta, Barcelona, 43592 Catalonia, Spain; ^5^UUDD Tortosa-Terres de l'Ebre, CAP Temple, EAP Tortosa Est, Institut Català de la Salut, 43500 Tortosa, Spain; ^6^EAP-Camarles-Aldea-Ampolla, Catalonian Health Institute, SAP Terres de l'Ebre, Health Department, Generalitat de Catalunya, CAP Camarles, 43894 Tortosa, Spain; ^7^EAP-Alcanar-St Carles de la Rápita, Catalonian Health Institute, SAP Terres de l'Ebre, Health Department, Generalitat de Catalunya, CAP St Carles de la Rápita, 43894 Tortosa, Spain

## Abstract

**Aims/Introduction:**

Determining the prevalence of diabetes and its cardiovascular complications and all-cause mortality in older chronic complex patients.

**Materials and Methods:**

We carried out a multicenter retrospective study and included a randomized sample of 932 CCP people. We assessed the prevalence of diabetes according to World Health Organization criteria. Data included demographics and functional, comorbidity, cognitive, and social assessment.

**Results:**

The prevalence of diabetes was 53% and average age 81.16 ± 8.93 years. There were no significant differences in the survival of CCP patients with or without DM, with or without ischaemic cardiopathy, and with or without peripheral vascular disease. The prognostic factors of all-cause mortality in patients with DM were age ≥ 80 years [HR 1.47, 95% CI 1.02–2.13, *p*  0.038], presence of heart failure [HR 1.73, 95% CI 1.25–2.38, *p*  0.001], Charlson score [HR 1.20, 95% CI 1.06–1.36, *p*  0.003], presence of cognitive impairment [HR 1.73, 95% CI 1.24–2.40, *p*  0.001], and no treatment with statins [HR 1.49, 95% CI 1.08–2.04, *p*  0.038].

**Conclusions:**

We found high prevalence of DM among CCP patients and the relative importance of traditional risk factors seemed to wane with advancing age. Recommendations may include relaxing treatment goals, providing family/patient education, and enhanced communication strategies.

## 1. Introduction

The World Health Organization [1] has reported an increase in the ageing population living with major chronic diseases such as diabetes, dementia, cardiovascular disease, and certain cancers, with most of the increase in developing countries. Health experts have called this phenomenon the “grey tsunami” [2] due to its impact on the health system. Type-2 diabetes is one of the most common chronic diseases affecting older people and its prevalence increases with age. It has been estimated that the number of people over 65 with diabetes will increase by 4.5-fold by 2050 [3]; and diabetes is linked to higher mortality, reduced functional status, impaired quality of life, increased risk of institutionalization [4], and mortality.

Several publications have described the spectrum of comorbidities and functional impairment in ageing populations [5–7]. They emphasize a number of key features such as the emergence of cognitive dysfunction and frailty that can worsen adverse outcomes of diabetes such as emergency visits, increased fall risk, and mortality. The main goal of this study was to determine the prevalence of diabetes and its cardiovascular complications and all-cause mortality in complex chronic patients.

## 2. Materials and Methods

### 2.1. Study Population and Data Collection

This cohort study (2013–2016) included 3,490 cases registered as* complex chronic patient* enrolled in a large, integrated health primary care teams in the* Terres de l'Ebre* health area in Catalonia (Spain) with a sampling frame that included a randomized sample of 932 members. The source population was identified from the Catalonian Health Institute Registry as of 1 January 2013 to 31 December 2014. We included subjects if they met at least four of the following* criteria*: (1) age (≥65 years old), (2) chronic comorbidities (≥4), (3) psychosocial disorders (cognitive impairment or psychological disorder with functional disability), (4) geriatric conditions such as functional disability (Barthel score < 55, living in assisted living, in nursing home, or with in-home caregivers) or recurrent falls or fall risk, (5) previous high healthcare use (two hospitalizations not programmed for exacerbation of chronic pathologies or three emergency department visits in the last year), (6) ≥4 active medications in the last 6 months, and (7) living alone or with a caregiver ≥ 75 years old. Established in January 2013, the registry is written, managed, and updated by the nursing service in primary care using the* Shared Individual Intervention Plan* [*pla d'intervenció individualitzat compartit *(*PIIC*)]. Follow-up of this cohort members was initiated on 1 January 2013, and individuals were censored at the first occurrence of death that had occurred from any cause or at the end of the study (30 September 2016).

### 2.2. Variables

We collected data on demographic characteristics and data related to clinical, functional, cognitive, and social assessment. Comorbid conditions are defined using standard outpatient and inpatient ICD-9 codes by electronic data capture including pharmacy records, laboratory data, and outpatient, emergency room, and hospitalization diagnoses across all primary care centers and hospital. Charlson comorbidity index, short version, was scored. Polypharmacy was defined as five or more daily medications. If there was a diagnosis of atrial fibrillation (AF), CHA_2_DS_2_VAS_C_HAS-BLED scores were included. Presence of cognitive impairment, a disease-specific diagnosis of cognitive impairment, without specification of subtype or severity, was measured using the Pfeiffer test. The variable definition includes recurrent falls or fall risk and presence of disability by Barthel score to assess dependence in ADL.

Currently, 82% of people registered as CCP have available clinical data in their PIIC report.

### 2.3. Criteria for the Definition of Diabetes

Diabetes (DM) was diagnosed according to any of the following WHO criteria: fasting plasma glucose (FPG) ≥ 7.0 mmol/l (126 mg/dl) or 75 g oral glucose tolerance test (OGTT) with FPG ≥ 7.0 mmol/l (126 mg/dl) and/or 2-hour plasma glucose ≥ 11.1 mmol/l (200 mg/dl) or HbA1c ≥ 6.5%/48 mmol/mol or random plasma glucose ≥ 11.1 mmol/l (200 mg/dl) in the presence of classical diabetes symptoms.

### 2.4. Statistical Analysis

Time to event analysis was performed using the Kaplan-Meier and Log Rank test. To estimate hazard ratios, mean survival time, and survival probabilities, we used a multivariate Cox regression. Multivariate Cox proportional hazards regression models were fitted to identify significant variables associated with the time to death since diagnosis as CCP. The adjusted model included the following baseline characteristics and the differences observed between DM and no DM and predictive factors for each event: age, sex, Charlson index, and factors in CHA_2_DS_2_VAS_C_ scales and active pharmacy. The analyses were performed using IBM SPSS version 19.0.

## 3. Results

The baseline characteristics of the CCP group are shown in Table 1. The prevalence of CCP was 1.94% in the total population and 7.01% in those ≥60 years old. Diabetes had been diagnosed in 53% of the CCP population, with an average age of 81.16 ± 8.93 years, significantly younger (*p* < 0.001) than those CCP without DM, but with a higher cardiovascular risk (*p*  0.015), a higher risk of stroke (*p* < 0.001), more chronic conditions (*p* < 0.001), and a higher number of prescribed drugs (*p* < 0.001), but a higher Barthel score (*p*  0.003) and lower prevalence of cognitive impairment (*p*  0.001).

The patients were divided into three major subgroups defined jointly by age (<70, 70–79, and ≥80 years) and prevalence of cardiovascular complications (Table 2). There was a steady increase in the prevalence of cardiovascular comorbidities such as atrial fibrillation and heart failure, cognitive impairment, loss of autonomy in basic daily activities, increased fall risk, and the all-cause mortality rates associated with ageing. Other traditional risk factors associated with diabetes such as hypertension, dyslipidemia, and macrovascular complications such as ischaemic cardiopathy and peripheral artery disease stayed the same or even decreased with ageing.

**Figure 1:**
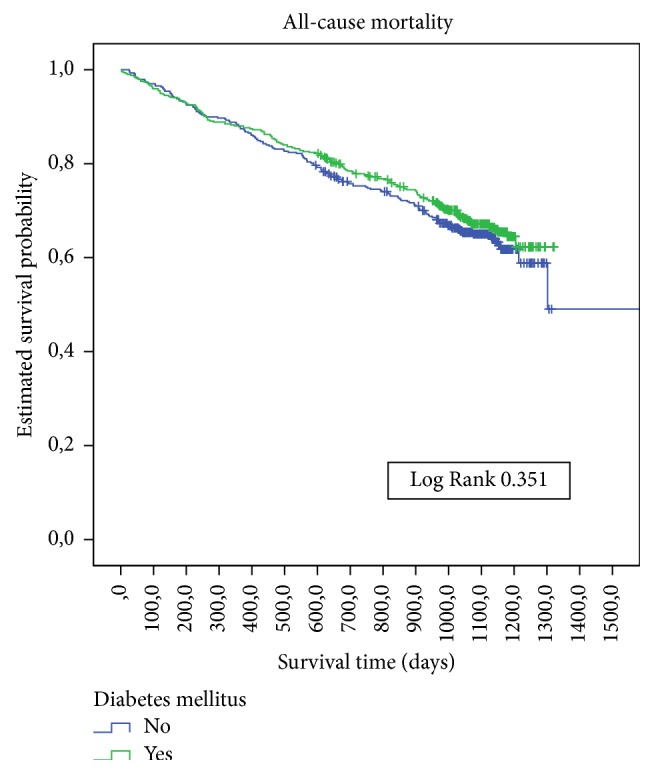
Kaplan-Meier estimates of survival during follow-up in CCP with or without diabetes mellitus at baseline.

**Figure 2:**
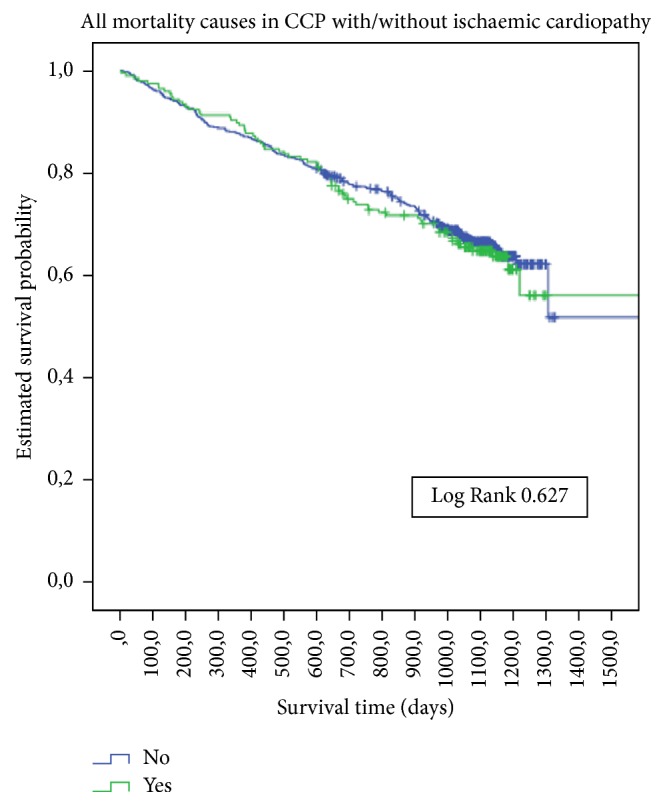
Kaplan-Meier estimates of survival during follow-up in CCP with or without ischaemic cardiopathy at baseline.

**Figure 3:**
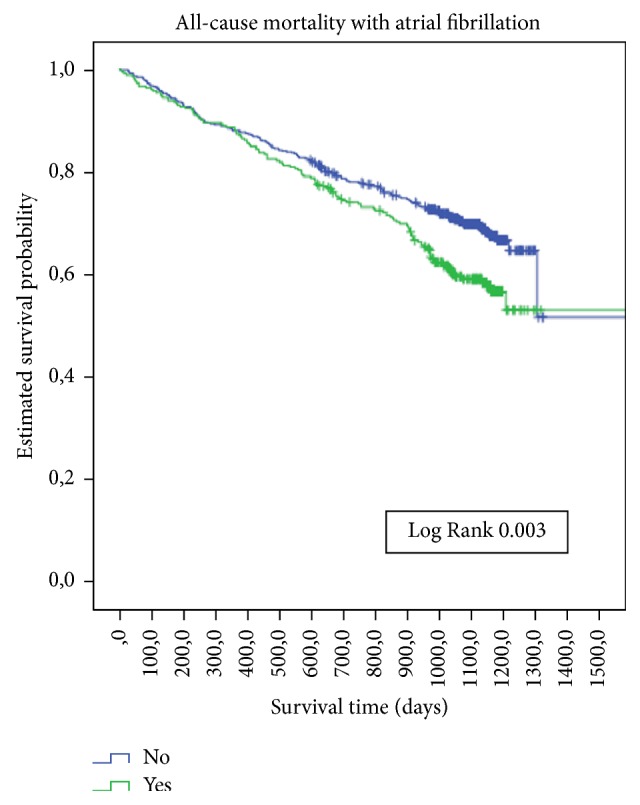
Kaplan-Meier estimates of survival during follow-up in CCP with or without atrial fibrillation at baseline.

**Figure 4:**
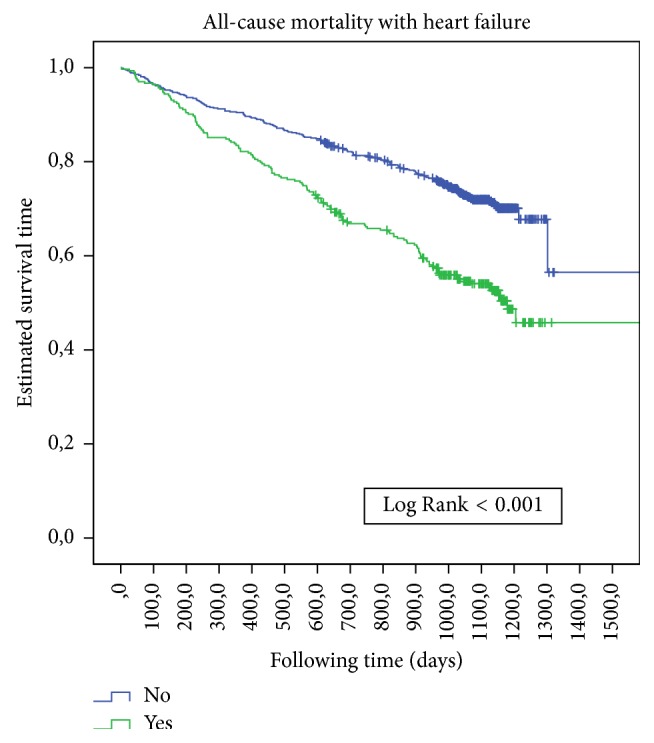
Kaplan-Meier estimates of survival during follow-up in CCP with and without heart failure at baseline.

The average follow-up time was 2.75 years (95% CI 2.42–3.07). The all-cause mortality rate was 32.8% in DM and 35.8% in non-DM patients. The incidence rate of death was 13.1/100 person-years in DM and 11.7/100 person-years in non-DM patients. There were no significant survival differences (Figure 1) between those with or without DM, with or without ischaemic cardiopathy (Figure 2), or with or without peripheral artery disease. There was a significant survival difference in the case of atrial fibrillation (*p*  0.003) (Figure 3) and heart failure (*p* < 0.001) (Figure 4).  Table 3 shows number of deaths by decade of age.

In the CCP population with DM the prognostic mortality factors identified by the multivariate method were age ≥ 80 years [HR 1.47, 95% CI 1.02–2.13, “*p*  0.038”], heart failure [HR 1.73, 95% CI 1.25–2.38, “*p*  0.001”], the Charlson score [HR 1.20, 95% CI 1.06–1.36, “*p*  0.003”], cognitive impairment [HR 1.73, 95% CI 1.24–2.40, “*p*  0.001”], and no treatment with statins [HR 1.49, 95% CI 1.08–2.04, “*p*  0.038”].

We can define an epidemiological model with a high prevalence of DM (53%) associated with classical risk factors such as hypertension (84.1%), dyslipidemia (52.1%), atrial fibrillation (37.9%), and stroke (28.3%) to which other risk factors can be added such as age ≥80 years (70.2%), cognitive impairment (44.1%), heart failure (35.6%), CNS depressant drugs (56.9%), fall risk (23.2%), and the lowest Barthel score (59, 95% CI 57.1–62.08), all of which increase the risk of mortality.

## 4. Discussion

The registered prevalence of CCP in our study was higher than in other developed countries [8] as was the prevalence of diabetes mellitus in our CCP population [2, 9, 10]. The frequency of comorbidity burden such as cognitive impairment, polypharmacy, functional disability of basic activities of daily living, and limited availability of caregiver support may be a substantial problem in implementing a management plan. A consensus has developed on how to treat older people with diabetes [11, 12], but given that complications may not be present or may take many years to develop, efforts should be adapted depending on the average remaining life expectancy and degree of impairment of quality of life.

The authors believe a strategic change will involve moving away from “*the diabetic patient with complications*” to “*CCP with chronic comorbidities.*” Therefore, while classically diabetes mellitus has been associated with the development of micro and macrovascular complications, with an ageing population, it is not yet clear whether the presence of chronic cardiovascular comorbidities or a higher risk of undergoing acute cardiovascular events (higher CHA_2_DS_2_VAS_C_ score) should be approached as preventable and modifiable risk factors (antiaggregant, statins) or as one more condition of the “chronic complex patient” that needs to be controlled but without benefits for cardiovascular risk.

Given that it is unknown how long the DM is running and that most risk score tables exclude 75% of our CCP due to their age, the use of such tables is pointless because we cannot relate the cardiovascular complication rates to a risk score. On the other hand, strategies to modify the incremental tendency for DM involving the promotion of healthy lifestyles (mainly associated with diet and physical activities) and the detection of high risk patients are difficult to apply at this vital stage. The priorities should be avoiding or lowering the risk of complications (hypoglycemia, hyperglycemia, fall, and polypharmacy) via an appropriate stratification using the functional autonomy based on adaptations to individual environments. The literature related to interventions for lifestyle diseases in developing countries is very limited, probably due to the multitude of possible health endpoints and interventions, the multiple sources of the problem, and the limited knowledge of means of changing individual and population behavior [13–15].

In contrast with other studies [16], our results show that the incidence of all-cause mortality and the prevalence of cardiovascular diseases do not differ significantly among CCP with and without DM. The data about deaths related to diabetes are confusing and come from different age cohorts: according to the World Health Organization, 43% of all deaths due to high blood glucose occur before the age of 70 [17]; and the proportion of deaths attributable to diabetes was estimated to be 11.5% in the United States [18, 19]. In our study the mortality rate due to diabetes was 17%, and <70 years old is when the difference in mortality is higher between DM and no DM. This fact supports our thought of a different epidemiologic profile in this group of population. However, we found risk factors associated with ageing such as cognitive impairment and heart failure that have been described previously as prognostic factors for mortality [5–7] in the CCP population. This supports the idea that all-cause mortality is more affected by ageing factors than by specific complications of DM. Likewise the incidence of DM in this CCP population may be more of a consequence of the ageing process than an independent disease. The comorbidities may drive all-cause mortality and the contribution of diabetes in the presence of complex chronic diseases to overall mortality seems to be only minimal.

Despite the above, we should not underestimate the importance of evaluating and treating cardiovascular risk given that it plays a key role in cardiovascular prevention in all national and international guidelines [20], mainly among patients with a high level of functional dependence. The relative importance of traditional risk factors seems to wane with advancing age [21] and for this reason; we emphasize the importance of redefining the care strategy and adapting it to comorbidities and functional autonomy rather than achieving excellent levels of glycated haemoglobin or total cholesterol.

The limitations of the present approach are related to the possibility of overestimating the prevalence of DM among the CCP population as a consequence of the definition criteria despite being a randomized sample; not knowing the duration of DM in order to prevent complications; and perhaps the frequency with which diabetes is listed as the underlying cause of death is not a reliable indicator of its contribution to the mortality profile. Also, we need to determine accurately whether diabetes was the underlying cause of death or was only an associated cause of death.

## Figures and Tables

**Table 1:** Baseline characteristics of CCP with and without diabetes.

CCP patients	No diabetes	Diabetes	*p*
*N* (%)	438 (47.00%)	494 (53.00%)	
Age (average ± SD)	84.22 ± 10.6	81.16 ± 8.0	<0.001
Percentage >80 years old *n* (%)	340 (77.6%)	315 (63.8%)	<0.001
Women *n* (%)	236 (53.9%)	252 (51.0%)	0.394
CCP criteria number (average ± SD)	3.85 ± 1.26	3.86 ± 1.12	0.955
Hypertension *n* (%)	362 (80.4%)	422 (85.4%)	0.044
Dyslipidemia *n* (%)	195 (44.5%)	326 (66.0%)	<0.001
Atrial fibrillation *n* (%)	176 (40.2%)	149 (30.2%)	0.002
Ischaemic cardiopathy *n* (%)	87 (19.9%)	109 (22.1%)	0.422
Peripheral artery disease *n* (%)	58 (13.2%)	91 (18.4%)	0.032
Chronic kidney insufficiency < 30 mlClCr	43 (9.8%)	73 (14.7%)	0.042
Heart failure *n* (%)	153 (34.9%)	150 (30.4%)	0.142
Charlson score (average ± SD)	2.05 ± 1.30	2.95 ± 1.30	0.015
Stroke before CCP *n* (%)	104 (23.7%)	96 (19.4%)	0.111
Stroke after CCP *n* (%)	36 (8.2%)	30 (6.1%)	0.249
CHA_2_DS_2_VAS_C_ score (average ± SD)	5.97 ± 2.38	7.14 ± 5.97	<0.001
HAS_BLED score (average ± SD)	2.88 ± 1.14	3.10 ± 1.05	0.077
Daily medications number (average ± SD)	7.8 ± 3.29	9.80 ± 3.60	<0.001
Polypharmacy ≥ 4 *n* (%)	392 (89.5%)	484 (98%)	<0.001
Polypharmacy ≥ 10 *n* (%)	142 (32.4%)	258 (52.2%)	<0.001
Cognitive impairment *n* (%)	179 (40.9%)	159 (32.2%)	0.006
Pfeiffer test score (average ± SD)	3.43 ± 3.39	2.72 ± 3.13	0.001
Barthel score (average ± SD)	62.8 ± 32.41	69.02 ± 31.28	0.003
Barthel score < 60 *n* (%)	182 (41.6%)	161 (32.6%)	0.005
Fall risk *n* (%)	103 (23.9%)	85 (17.2%)	0.018
Gijón score (average ± SD)	9.53 ± 5.12	10.46 ± 3.87	0.363
Antiaggregant treatment *n* (%)	147 (33.6%)	238 (48.2%)	<0.001
Anticoagulant treatment *n* (%)	147 (33.6%)	123 (24.9%)	0.003
Statin treatment *n* (%)	149 (34.0%)	274 (55.5%)	<0.001
Proton pump inhibitor treatment *n* (%)	285 (65.1%)	358 (72.5%)	0.016
Selective serotonin reuptake inhibitors (SSRIs) *n* (%)	131 (29.9%)	149 (30.2%)	0.943
CNS depressant drugs *n* (%)	249 (56.8%)	265 (53.6%)	0.356
Death *n* (%)	157 (35.8%)	162 (32.8%)	0.334

**Table 2:** Baseline characteristics of CCP patients according to age.

CCP patients	<70 years old	70–79 years old	≥80 years old
*N* (% all)	93 (9.97%)	184	655
Age (average CI 95%)	61.39 (59.6–63.1)	75.6 (75.2–76.02)	87.5 (87.2–87.9)
Women *n* (% group)	41/93 = 44.08%	82 (44.6%)	365 (55.7%)
CCP criteria number (average CI 95%)	2.9 (2.7–3.22)	3.69 (3.5–3.8)	4.02 (3.94–4.12)
Diabetes mellitus *n* (% group)	53 (57.%)	125 (67.9%)	16 (48.2%)
Hypertension *n* (% group)	63 (67.7%)	160 (87.0%)	551 (84.1%)
Dyslipidemia *n* (% group)	55 (59.1%)	125 (67.9%)	341 (52.1%)
Atrial fibrillation *n* (% group)	17 (18.3%)	60 (32.6%)	248 (37.9%)
Ischaemic cardiopathy *n* (% group)	20 (21.5%)	43 (23.4%)	133 (20.3%)
Peripheral artery disease *n* (% group)	17 (18.3%)	41 (22.3%)	91 (13.9%)
Chronic kidney insufficiency < 30 mlClCr *n* (% group)	12 (12.9%)	24 (13.1%)	80 (12.2%)
Heart failure *n* (% group)	17/93 = 18.27%	53 (28.8%)	233 (35.6%)
Charlson score (average CI 95%)	2.17 (1.87–2.47)	2.7 (2.5–2.9)	2.52 (2.42–2.63)
Stroke *n* (% group)	21 (22.6%)	51 (27.8%)	185 (28.3%)
CHA_2_DS_2_VAS_C_ score (average CI 95%)	4 (3.11–4.9)	5.17 (4.84–5.51)	5.03 (4.88–5.20)
Stroke risk/year (average CI 95%)	4.78 (3.55–6.02)	6.81 (6.2–7.4)	6.57 (6.27–6.87)
Daily medications number (average CI 95%)	9.21 (8.37–10.6)	10.1 (9.5–10.53)	8.5 (8.24–8.77)
Cognitive impairment *n* (% group)	14 (15.1%)	35 (19.0%)	289 (44.1%)
Pfeiffer test score (average CI 95%)	1.40 (0.86–1.95)	1.53 (1.19–1.89)	3.71 (3.46–3.97)
Barthel score (average CI 95%)	82.8 (77.3–88.4)	80.67 (76.9–84.4)	59. (57.1–62.08)
Fall risk *n* (% group)	5 (5.4%)	31 (16.8%)	152 (23.2%)
Antiaggregant treatment *n* (% group)	30 (32.3%)	82 (44.6%)	273 (41.7%)
Anticoagulant treatment *n* (% group)	25 (26.9%)	55 (29.9%)	175 (26.7%)
HAS_BLED (average CI 95%)	2.93 (2.22–3.65)	3.06 (2.81–3.31)	2.96 (2.82–3.11)
Bleeding risk/year (average CI 95%)	5.18 (3.0–7.37)	4.89 (4.0–5.8)	4.95 (4.5–5.41)
Statin treatment *n* (% group)	54 (58.1%)	118 (64.1%)	251 (38.3%)
CNS depressant drugs *n* (% group)	44 (47.3%)	97 (52.7%)	373 (56.9%)
Death *n* (% group)	12 (12.9%)	48 (26.1%)	259 (39.5%)
Average follow-up time *n* (days CI 95%)	1348 (719–1977)	947 (901–993)	971 (825–1117)

**Table 3:** Number of deaths according to age.

Age	Diabetes *N* (%)	No diabetes *N* (%)	*p*	All
<70 (*n* 93)	9 (17.0%)	3 (7.5%)	0.150	12 (12.9%)
70–79 *n* (184)	32 (25.6%)	16 (27.1%)	0.480	48 (26.1%)
80–89 *n* (435)	74 (31.6%)	72 (35.8%)	0.361	146 (33.6%)
≥90 *n* (220)	47 (57.3%)	66 (47.8%)	0.209	113 (51.4%)
Total (*n* 932)	162 (32.8%)	157 (35.8%)	0.181	319 (34.2%)
